# Distinctive Spatial and Laminar Organization of Single Axons from Lateral Pulvinar in the Macaque

**DOI:** 10.3390/vision4010001

**Published:** 2019-12-18

**Authors:** Kathleen S. Rockland

**Affiliations:** Department of Anatomy & Neurobiology, Boston University School of Medicine, Boston, MA 02118, USA; krock@bu.edu

**Keywords:** core and matrix, cortical layers, divergence, driving, extrastriate cortex, feedforward, multiple scales, multi-specific thalamo-cortical

## Abstract

Pulvino-cortical (PC) projections are a major source of extrinsic input to early visual areas in the macaque. From bulk injections of anterograde tracers, these are known to terminate in layer 1 of V1 and densely in the middle cortical layers of extrastriate areas. Finer, single axon analysis, as reviewed here for projections from the lateral pulvinar (PL) in two macaque monkeys (*n* = 25 axons), demonstrates that PL axons have multiple arbors in V2 and V4, and that these are spatially separate and offset in different layers. In contrast, feedforward cortical axons, another major source of extrinsic input to extrastriate areas, are less spatially divergent and more typically terminate in layer 4. Functional implications are briefly discussed, including comparisons with the better investigated rodent brain.

## 1. Introduction

The visually-related pulvinar in nonhuman primates (NHP) is a complex nucleus consisting of multiple retinotopically organized subnuclei, each with a mix of morphologically and neurochemically distinct cell types [1,2,3,4] and a “complex mesh of interconnections” [5]. Pulvino-cortical (PC) projections have repeatedly been shown to impact on both sensory and higher order visual processing. Deactivation of the lateral pulvinar (PL) eliminated visual responsivity in the upper layers of area V1 [5] in galago, augmented or reduced responses in area V2 [6] in cebus, and resulted in multiple changes in areas V4 and IT, consistent with a role in normal attention and sensory processing [7] in macaque.

Visual processing in occipitotemporal areas is generally thought to involve interactions of pulvino-cortical (PC) projections with extrinsic and intrinsic cortico-cortical (CC) connections. While detailed microcircuitry data will be necessary to further understand these interactions, experiments in galago have identified several functionally relevant comparisons of the V1 and PL projections to V2, in that these appear to target similar layers and both lack parvalbumin. Projections from the PL to V2, however, had larger boutons, suggestive of greater synaptic efficacy [8].

In this brief review, I emphasize results at single axon, Golgi-like resolution of PL axons in comparison with CC axons in early visual areas V1, V2, V4, and area MT (middle temporal, or V5) of NHP. This finer level of resolution demonstrates that PL axons in these areas are morphologically distinct from CC in arbor number, arbor size, and overall spatial topology, as well as in laminar organization. For more extensive reviews of pulvinar organization and connectivity, see [9,10,11,12,13,14].

Results are largely based, except for V1, on serial reconstructions of 25 axons anterogradely labeled by tracer injections in the PL at the lateral border of the nucleus, near the representation of horizontal meridian [15] (*n*= two monkeys). CC comparisons are drawn from a series of similar studies of anterogradely labeled single axons after tracer injections in V1, V2, or V4 [16,17,18,19]. As stated in the original publications, no correction was made for shrinkage during histological processing. Note, however, that similar perfusion and tissue processing methods were used across the studies.

## 2. PC Axons are Spatially Divergent

### 2.1. Area V1

PC axons to V1 preferentially target layer 1 (see below), where they form a plexus generally parallel to the pia [5,15]. The spatial extent of individual axons is unknown, but 0.35–0.50 µm segments are commonly found in a focus of terminations (Figure 5 in [15]). These are suggestive of a wider divergence (>1.0 mm), as in fact tends to be characteristic of terminations in layer 1; that is, feedback CC axons in V1 [19] and lateral geniculate (LG) inputs to layer 1 are widely divergent [20].

A comparison of input to mouse V1 from LG and lateral posterior nuclei (LP) concludes that LG input to layer 1 is topographically organized but input from LP is only coarsely topographic, with much larger receptive fields and it originates from widely dispersed locations in the visual field. This contrast was interpreted to suggest that LP inputs provide contextual information about the visual scene, with spatial extension beyond the strong retinotopic bias of local V1 neurons: “LP inputs may therefore contribute to surround modulation of V1 neurons, or to state-dependent or behavioral modulation of visual responses across visual space” [21]. In galago, more specific effects have been demonstrated by manipulations of PL inputs to V1: reversible inactivation prevented supragranular neurons in V1 from responding to visual stimulation, while focal excitation of the PL increased by four-fold the visual responses in retinotopically coincident V1 [5]. Excitation of the PL after lesions of LG successfully activated supragranular neurons in V1 [5].

### 2.2. Areas V2 and V4

In our material, PL axons had more spatially separate arbors that diverged over a larger cortical territory than the CC axons terminating in the same area. In area V2, most V1 axons had single arbors, less than 0.4 mm in diameter; and those with multiple arbors formed patches over a hollow domain of about 1.2 mm × 0.3 mm [16]. In area V4, most axons from V2 had two to four arbors, spatially distributed over larger territories, but typically less than 2.0 mm × 1.25 mm (19 of 20 axons in [18]).

In contrast, reconstructed PC axons in V2 distributed over a territory greater than 2.0 mm, and those in V4, over an even larger territory (*n* = 2 axons, 3.0 × 2.6 mm, and 2.0 × 1.25 mm). One of the PL axons in V4 had at least eight smaller collaterals in addition to two arbors in layer 4. This complex, mixed topology—terminal clusters together with linear segments—has been reported for a V4 feedback axon terminating in V1 (Figure 12 in [22]) and for the intrinsic collateral tree of a layer 3 pyramidal neuron in cat visual cortex [23]. In the latter instance, the clustered collaterals, but not the linear, were myelinated along the preterminal extent within the gray matter.

Why PL arbors extend over wider territories in V2 and V4 than feedforward CC in the same target area is unclear. A straightforward explanation might be the apparently larger receptive fields of PL neurons [21]. Given that factor, a conservation of retinotopic match in relation to the target cortex might result in an apparently wider axonal spread. Another factor might be the particular kind of functional specialization of PL vs. CC neurons, along the lines of the compact arbors associated with parvocellular LG axons in V1 vs. the more distributed magnocellular LG axons [20,24]. The different spatial scale of PL and CC axons in these areas raises the possibility of multiple or partially overlapping functional maps, not unlike the nested maps in area V1 for ocularity, orientation, color, and spatial frequency.

A differential relationship to cortical modularity, notably the cytochrome oxidase (CO) compartments in area V2 should also be considered. Bulk injections of WGA-HRP in the PL produce stripelike terminations coinciding with the CO compartments in V2 [25], and the connections from V1 to V2 are also broadly organized with respect to CO domains [26]. Less clear, however, is how individual axons, either the CC or PL, are oriented within or across stripes, and whether and how these converge on common or distinct postsynaptic neurons. Similar questions arise about a convergence of PL axons and the patchy distribution of pyramidal cell intrinsic collaterals, which form terminal patches (“daisy”) estimated as radiating 2.0–3.0 mm from a given cell body [23,27].

### 2.3. A Smaller Scale in Area MT

Only a few axons from the PL (*n* = 6) have been reconstructed in area MT [15]. Curiously, five of these had small arbors (<0.2 mm wide), smaller than the PL arbors in V2 and V4. This is also smaller than V1 axons terminating in the middle layers of area MT, although about comparable to the small V1 collaterals in layer 6 of area MT [17]. The apparent size progression of arbors from one injection site in the PL (V4 > V2 > MT) might reflect different PL subpopulations within the injection site. These are known to include giant calbindin-positive neurons, non-giant calbindin-positive neurons, and parvalbumin positive neurons (reviewed [2,8,28,29]). Alternatively, smaller arbors might be collateralized projections from other areas. Further investigation with a larger sample size is needed.

## 3. PC Axons Terminate in Multiple Layers

### 3.1. Areas V2, V4, and MT

In the early visual cortical areas V2 and V4, feedforward CC axons preferentially target the middle layers 3 and/or 4, sometimes with collaterals in layer 5 [16,18,27]. Area MT is an exception, where axons from V1, but not those from V2, have one to two small arbors in layer 6 in addition to what are typically two larger arbors in the middle layers [17].

**Figure 1:**
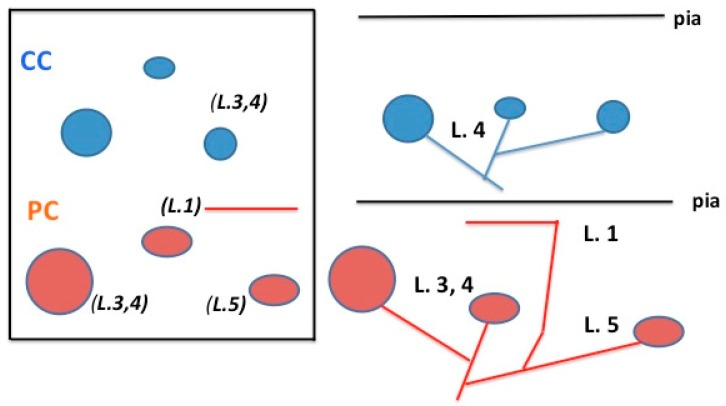
Schematic representation comparing typical spatial and laminar organization of cortico-cortical (CC) (in blue) and pulvino-cortical (PC) (in red) axons for areas V2 and V4. In the tangential view (at left), looking down at the cortical surface, the CC and PC axons both have three arbors, but these are distributed over different spatial extents. A layer 1 collateral is shown for the PC axon. For 3-D orientation, layers are indicated in italics and parenthesis. This is re-mapped in a coronal view (at right), where the different laminar organization is more evident. See text for further details. L = layer.

A single PL axon, terminating in V2 or V4, commonly has one or several arbors in layers 3 and/or 4, defined as “main arbor” on the basis of larger size and greater number of boutons. In area V2, additional arbors variably occur in layers 1, 5, and/or 6. Of seven PL axons terminating in the lunate sulcus, four had collaterals in layer 1. Of nine axons terminating in inferior occipital sulcus, only one had collaterals in layer 1, and the others had small collaterals in layers 5 and/or 6. This strongly contrasts with the organization of CC projections to these areas, where terminations outside the middle layers are not usual. Moreover, the arbors of a single PL axon are spatially distributed and offset, rather than vertically aligned (see Figure 1).

Two axons reconstructed from the PL to V4 each had two arbors in layers 3 and 4, with smaller collaterals spatially separate in layer 5 (Figures 16 and 17 in [15]). Axons in the superior temporal sulcus (likely area MT) had two to three small arbors in the middle layers (layer 3 and adjoining layer 4). Of these, four had collaterals in layers 5 and/or 6. One apparently terminated only in layer 6.

Interestingly, there is some indication that CC projections in association cortical areas may also have a spatially distributed, multi-laminar termination pattern (Figure 3 in [30]). In both cases (PL and CC), such a pattern would seem to indicate a differential recruitment, on the part of the spatially distributed arbors, in the composition and proportions of postsynaptic neuron types and the postsynaptic dendritic locations. In the context of feedforward/feedback connections, a multi-laminar termination pattern might suggest a feedforward influence in one cortical locus, but a feedback, layer 1 influence in the same area, although spatially offset.

There is physiological evidence in mice that that thalamo-cortical inputs from the posterior medial nucleus (POm) have layer-specific functional effects in relation to pyramidal cells in layer 2 (weak, delayed, thalamus-evoked inhibition) vs. those in the deeper layers [31].

Multi-laminar thalamo-cortical (TC) terminations have been observed from the medial geniculate body to auditory cortex of the macaque [32]. In this system, several laminar termination patterns have been distinguished (layer 1 alone, layer 4 alone, or multiple layers) and thought to reflect heterogeneous, neurochemically differentiated thalamic populations. The observation that a single thalamic axon can terminate in multiple layers, including both layers 1 and 4, gives a more nuanced perspective on the idea of core vs. matrix TC projections (respectively, targeting layers 4 or 1), which has come to be often stated as a crisp dichotomy (reviewed [2,11,12]).

### 3.2. Area V1

PL axons projecting to area V1 terminate only or mainly in layer 1 (reviewed in [5,15,25]). This might be taken to indicate that PL terminations have functionally significant, preferential postsynaptic targets in layer 1, presumably distal apical dendrites and/or possibly specific inhibitory interneurons. There may also be an important convergence and interaction with other inputs in layer 1, including amygdalo-cortical [33], feedback cortical [19,22,27], and inhibitory inputs from zona incerta [34].

### 3.3. Collateral Branching to Areas V1 and V2

Injections of double retrograde tracers in V1 and V2 result in a proportion of double labeled neurons in the inferior and lateral pulvinar (from 5%–12%) [35]. This is somewhat surprising, since, as described above, PL connections in area V1 terminate preferentially in layer 1, but those in V2 occur in multiple layers. No PL axon has been reported, so far, that terminates only in layer 1 of V2 or V4. This dual termination pattern of a single axon in primary and extrastriate areas recalls the “multi-specific” category of TC axons, identified in rodents that terminate in multiple areas with different spatial and laminar patterns [36,37]. These have been overlooked in NHP; but at least for the visual system, our results show that branches of the same axon would be primarily “core-like” in V2 and “matrix-like” in V1 (and see [32]).

## 4. Quantitative Parameters: Bouton Size, Density, Number

Quantitative bouton features, as detected at the light microscopic level, can be a useful clue to synaptic strength and efficacy. In the geniculo-cortical pathway, for example, magnocellular axons have larger boutons than the parvocellular [20,24]. Another striking example are the two classes of cortico-pulvinar neurons, where small and large boutons (class I and II, respectively) have been associated with several other distinguishing features and identified with modulatory or “driving” influences ([11,12,29] and see discussion in [38]).

On the subject of connectional strength, there are several relevant recent studies in rodents. In mouse somatosensory cortex, for example, there is evidence that higher-order thalamic inputs from POm are stronger, with longer lasting effects, than cortical inputs [39]. Another study reports that axon terminals from PO are significantly larger in motor cortex than somatosensory, with markedly different laminar distribution and receptor mechanisms, likely in support of area-specific differences in functional impact [40]. A third study in extrastriate cortex of mice reports postsynaptic cell-type specificity of PC terminations, where cortico-striatal and cortico-amygdala neurons, followed by V1-projecting neurons are most responsive to pulvinar input in mice, and cortico-collicular projecting neurons, least responsive [41].

Comparable physiological data for CC and PC connections are not available in NHP; but several relevant morphological differences have been observed. In area V2 of bush baby [8], PC boutons (*n* = 87) were found to have a mean area of 0.47µm^2^ in contrast with those from area V1 (*n* = 88), with a mean area of 0.36 µm^2^. By comparison, geniculo-cortical terminations were measured as having a mean area of 0.70 µm^2^ (magnocellular) or 0.52 µm^2^ (parvocellular). In the macaque, a subset of larger PL boutons (diameter = 1.0–2.0 µm) was found intermingled within a dense PL focus in V2 [15]. One might speculate that these correspond to the subset of giant calbindin-positive PL neurons [28]; but these observations need to be extended to larger sample size; and, for judgments on putative efficacy, more data are needed on postsynaptic identity, dendritic targets, and functional influence.

The density of PL boutons has been reported as less than that of CC, as compared focus-to-focus (PL vs. CC) within V2 (Figure 4 in [15]). This might indicate a greater convergence of CC axons; but alternatively, since injection sites were not normalized across cases, could also be a consequence of differences in the injections or other individual variability in the experiments.

On the basis of available data, PL axons (Table 1 in [15]) appear to have a greater number of boutons than V1 CC axons projecting to V2 (Table 1 in [16]), than V2 axons projecting to V4 (Table 1 in [18]), or V1 axons projecting to area MT (Table 1 in [19] and Table 1 above). From this, one might infer that PL axons contact more postsynaptic neurons or that there are more synaptic contacts per neuron. By comparison, bouton counts for two parvocellular LG axons in layer 4 yielded 1520 and 1380, and 3200 for one magnocellular axon [24]. The number of boutons per individual axon, however, was markedly greater for the two PL axons reconstructed in area V4 (2110 and 5213), and markedly less for three of the axons terminating in the superior temporal sulcus (per axon: 222, 232, and 130 boutons).

Intrinsic pyramidal cell collaterals and feedback CC projections to V1 and V2 typically have a large number of boutons, exceeding 1000 boutons per neuron [19,23].

## 5. Open Questions

In summary, as briefly reviewed here, cortically projecting axons from PL evince a variety of phenotypes in terms of laminar and spatial distribution. Those projecting to area V1 preferentially target layer 1 and are hypothesized to be widely divergent, those to areas V2 and V4 terminate in multiple layers with spatially distributed arbors, and, curiously, at least some of the PL axons terminating in area MT form small, relatively compact arbors. These results, however, are based on two injection sites at the lateral edge of PL. A larger sample size is necessary to establish whether the apparent variability (especially across areas V2, V4, and MT) might derive from neurochemical or other cell-type specific properties of PL neurons and, importantly, to assess to what extent neurons at different retinotopic locations or in different visual pulvinar nuclei (inferior pulvinar) exhibit similar properties.

At the microcircuitry level, there are multiple questions in need of further investigation.

1. What is the synaptic convergence of PC and CC axons (in terms of synaptic size, synaptic numbers and cell-type and dendritic targets), especially in relation to the multi-laminar arbors reviewed here for PL axons?
2. How do PC axons interact with other systems: the numerically dense intrinsic collaterals of pyramidal cells, feedback projections in layer 1, and the various inhibitory populations?
3. What is the composition of neuron ensembles postsynaptic to the different arbors of a given axon? Is this related to finer modularity within the target area, at least for V2 and V4?
4. How do PL and other visual PC axons compare, structurally and functionally, with homologous thalamic terminations in other less visual species, such as rodents?

## Figures and Tables

**Table 1:** Number of boutons of lateral pulvinar (PL) axons compared with three CC projections.

	Range per Arbor	Average per Arbor	Range per Axon	Average per Axon
V1 to V2 [16]	37–308	123 (*n* = 32)	n.a.	n.a.
V2 to V4 [18]	n.a.	n.a.	107–858	438 (*n* = 14)
V1 to MT [19]	n.a.	n.a.	220–624	394 (*n* = 9)
PL to V2 [15]	130–410	253 (*n* = 8)	181–1394	578 (*n* = 12)

n.a.: data not available.
